# A symphony of anomalies: Isolated pulmonary artery agenesis meets congenital lung hypoplasia

**DOI:** 10.1002/ccr3.8645

**Published:** 2024-03-08

**Authors:** Nida Ansari, Sacide S. Ozgur, Alan Alcantara, Yasmeen Sultana

**Affiliations:** ^1^ Department of Internal Medicine St. Joseph's University Medical Center Paterson New Jersey USA

**Keywords:** absent pulmonary artery, congenital, pulmonary artery agenesis, pulmonary hypoplasia

## Abstract

Isolated agenesis of pulmonary arteries with congenital lung hypoplasia is rare. It can be found in childhood or adulthood if asymptomatic. We present a patient with congenital right lung hypoplasia with an absent right pulmonary artery.

## 1 INTRODUCTION

Isolated agenesis of pulmonary arteries with congenital lung hypoplasia is rare. It can be found in childhood or adulthood if asymptomatic. We present a patient with congenital right lung hypoplasia with an absent right pulmonary artery.

## 2 CASE HISTORY/EXAMINATION

A 24‐year‐old female with a past medical history of intermittent asthma, right lung hypoplasia, and dextrocardia presented to the Emergency Department for evaluation of shortness of breath. Her initial vitals showed a blood pressure of 118/87 mmHg, heart rate of 96 beats per minute, respiratory rate of 20/min, temperature of 36.2°C, and oxygen saturation of 96% on 5 L/min oxygen on nasal canula.

## 3 METHODS

**FIGURE 1:**
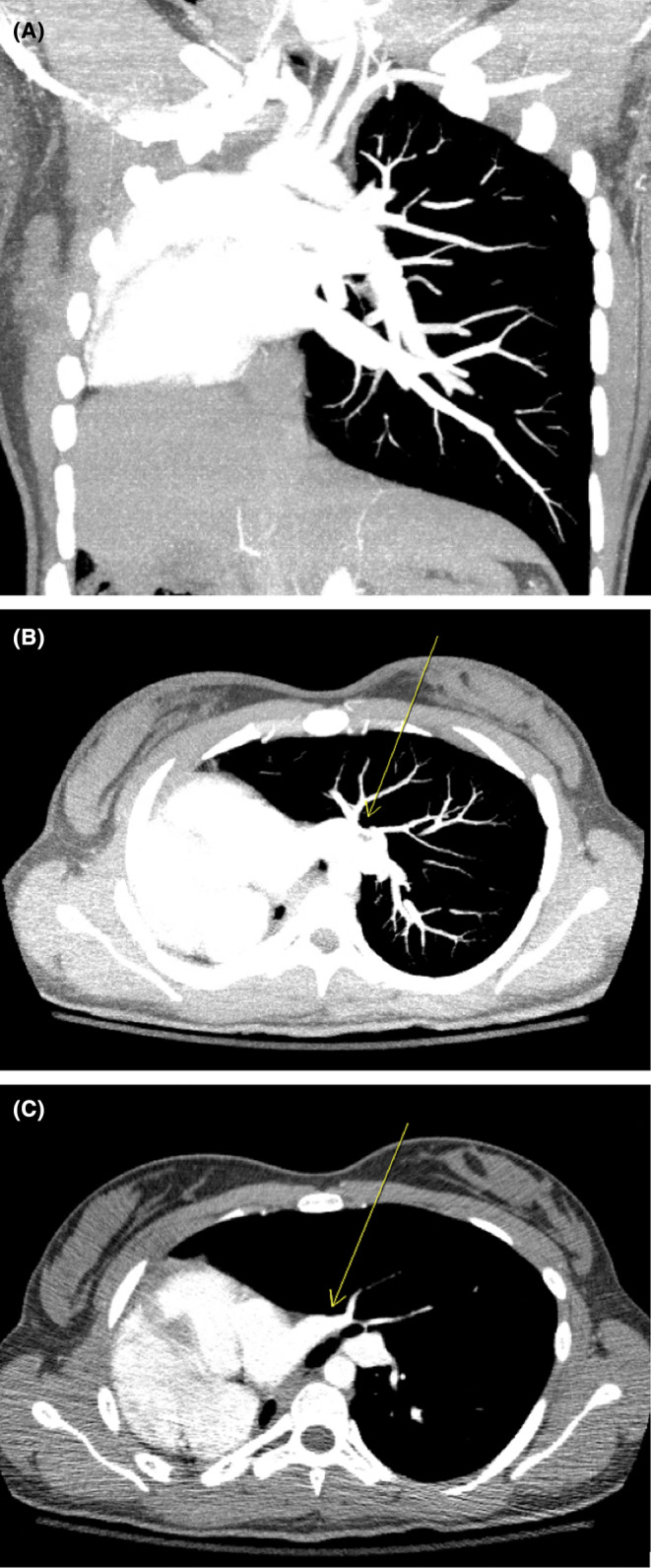
(A) Coronal view of computer tomography angiography of the chest demonstrating absent pulmonary artery, pulmonary hypoplasia, and dextrocardia. (B, C) Axial views of computer tomography angiography of the chest demonstrating singular pulmonary artery, absent pulmonary artery, pulmonary hypoplasia, and dextrocardia.

Upon evaluation via imaging, she was noted on computer tomography angiogram (Figure 1A–C) to have congenital hypoplasia of the right lung, isolated dextrocardia, and absent right pulmonary artery and its branches. Transthoracic echocardiogram was done which did not show any pulmonary hypertension or additional cardiac anomalies. It was concluded that her shortness of breath was secondary to an asthma exacerbation. The patient shortness of breath and oxygen requirement prompted admission to the critical care unit, where she was treated with intravenous steroids and respiratory treatments.

## 4 CONCLUSION

She ultimately improved and was transferred to the medical floors and shortly discharged afterward.

## 5 DISCUSSION

Congenital, isolated unilateral agenesis of pulmonary arteries (UAPA) is a developmental malformation that shows a unilateral absence of the pulmonary artery without the presence of a congenital heart disease.^1^ This often leads to pulmonary hypoplasia of the ipsilateral lung.^1^ UAPA is often classified as congenital or acquired.^1^ Congenital is defined as a developmental defect or malformation during embryogenesis.^1^, ^2^ Acquired UAPA due to chronic subclinical infection, typically secondary to tuberculosis, that involves the hilum of the lung.^1^ UAPA has a bimodal distribution in regard to age of presentation.^1^ Those who are present in childhood are often symptomatic as a result of congestive heart failure or pulmonary hypertension.^1^ Those who present as an adult are often asymptomatic, typically due to collateral vasculature to the affected lung.^1^, ^2^ While there are many modalities that can be used for diagnosis, CT or magnetic resonance pulmonary angiography is considered the gold standard.^1^ Management for UAPA is dependent on symptomology.^1^ Those who are non‐symptomatic or have minimal symptoms, often do not require treatment, and will require echocardiogram screening.^1^ Those with symptoms will require treatment with typically surgery if they have severe pulmonary hypertension; however, they can be treated with medications.^1^

## AUTHOR CONTRIBUTIONS

**Nida Ansari:** Conceptualization; data curation; formal analysis; funding acquisition; investigation; methodology; project administration; resources; software; supervision; validation; visualization; writing – original draft; writing – review and editing. **Sacide S. Ozgur:** Conceptualization; data curation; formal analysis; funding acquisition; investigation; methodology; project administration; resources; software; supervision; validation; visualization; writing – original draft; writing – review and editing. **Alan Alcantara:** Conceptualization; data curation; formal analysis; funding acquisition; investigation; methodology; project administration; resources; software; supervision; validation; visualization; writing – original draft; writing – review and editing. **Yasmeen Sultana:** Investigation; supervision.

## CONFLICT OF INTEREST STATEMENT

The authors report no conflict of interest. An ethical review is not necessary because this is a case report. This research received no specific grant from funding agencies in the public, commercial, or not‐for‐profit sectors.

## CONSENT

As this is a case report, consent was obtained for the purpose of this paper. Written informed consent was obtained from the patient to publish this report in accordance with the journal's patient consent policy.

## Data Availability

Data availability is not applicable to this article as no new data were created or analyzed in this study.
